# Using a Mixed IRT Model to Assess the Scale Usage in the Measurement of Job Satisfaction

**DOI:** 10.3389/fpsyg.2016.01998

**Published:** 2017-01-04

**Authors:** Tanja Kutscher, Claudia Crayen, Michael Eid

**Affiliations:** Department of Education and Psychology, Freie Universitaet BerlinBerlin, Germany

**Keywords:** job satisfaction, rating scale, large number of response categories, scale usage, response style, mixed IRT models

## Abstract

This study investigated the adequacy of a rating scale with a large number of response categories that is often used in panel surveys for assessing diverse aspects of job satisfaction. An inappropriate scale usage is indicative of overstraining respondents and of diminished psychometric scale quality. The mixed Item Response Theory (IRT) approach for polytomous data allows exploring heterogeneous patterns of inappropriate scale usage in form of avoided categories and response styles. In this study, panel data of employees (*n* = 7036) on five aspects of job satisfaction measured on an 11-point rating scale within the “Household, Income and Labor Dynamics in Australia” (wave 2001) were analyzed. A three-class solution of the restricted mixed generalized partial credit model fit the data best. The results showed that in no class the 11-point scale was appropriately used but that the number of categories used was reduced in all three classes. Respondents of the large class (40%) appropriately differentiate between up to six categories. The two smaller classes (33 and 27%) avoid even more categories and show some kind of extreme response style. Furthermore, classes differ in socio-demographic and job-related factors. In conclusion, a two- to six-point scale without the middle point might be more adequate for assessing job satisfaction.

## Introduction

Job satisfaction is a relevant indicator of quality of life and as such is well investigated in organizational contexts. As (Spector, 1997, p. vii) pointedly put it, “Job satisfaction is the degree to which people like their jobs.” More precisely, the term includes subjective evaluations of relevant work aspects and the affective states the person is experiencing while at work. Job satisfaction has become important in human resource management, guiding corporation policies in shaping processes and improving effectiveness. High job satisfaction is thought to reflect a good fit of employees' professional and personal characteristics to the job tasks and exhibits a positive effect on commitment and productivity (Judge et al., 2001). For individuals, high job satisfaction often implies an adequate work-life balance, which in turn increases well-being and life satisfaction (Kossek and Ozeki, 1998).

Because of its importance, a single-item measure of general job satisfaction is often included in national panel surveys, sometimes backed by measures for satisfaction with certain aspects of the job, such as income and relations with colleagues. What is striking is the diversity of response formats across studies. The number of response categories of the rating scale varies considerably: Only four categories were used in the Survey of Health, Ageing and Retirement in Europe (SHARE) as opposed to 11 categories in the German Socio-Economic Panel (GSOEP), the Household, Income and Labor Dynamics in Australia Survey (HILDA), and the Swiss Household Panel (SHP). Such a high number of response categories (a long response format) is intended to lead to a measure that reflects the fine-grained differences between subjects in the rated characteristic (Preston and Colman, 2000). However, it is unclear whether the ratings elicited by a long response format can be thought of as representative of the underlying job satisfaction or whether other processes shape the differences found in measure.

### Inappropriate scale usage

Answers to a survey questionnaire are based on individual's knowledge of the topic and his or her habit of thinking in a certain number of subjectively meaningful categories, for example, black and white thinking vs. sophisticated thinking (Viswanathan et al., 2004; Naemi et al., 2009). Response formats with very few response categories may not allow for sufficient differentiation, while response formats with very many categories may overburden subjects (Weng, 2004). Too few as well as too many response categories are therefore a potential source of inappropriate scale usage and bias. Inappropriate scale usage (ISU in the following) refers to individual tendencies in responding unrelated to the content of the question at hand (Paulhus, 1991). In general, simplifying strategies are frequently employed. Pronounced simplifying strategies are commonly known as response styles: the preference for extreme categories (extreme response style, ERS), preference of the middle category (MRS), as well as an acquiescent response style (ARS) and a disacquiescent response style (DARS). Empirically, these are found in major sample portions, for example, for ERS 25–30% of respondents, for MRS 11–33%, and for ARS 32–52% (Meiser and Machunsky, 2008; Carter et al., 2011; Wetzel et al., 2013). Less pronounced strategies such as avoidance of certain categories have received less attention (see for a recent overview Viswanathan et al., 2004; Van Vaerenbergh and Thomas, 2013). Eid and Rauber (2000) report that roughly a third of employees in their sample was using only five of the six presented response categories when asked to rate satisfaction with their superior. If such a misfit between the presented and the subjectively meaningful number of response categories exists, the scale will not adequately reflect the continuous underlying trait and hence violate assumptions for a rating scale (Meiser and Machunsky, 2008). Empirical results of the effects of length of the response format on scale usage behavior have revealed three important aspects:

1. There is interindividual heterogeneity in scale usage (Jin and Wang, 2014). Studies employing mixed IRT models mostly used a 4- to 6-point rating scale and often report at least two latent classes of individuals that differ in scale usage, independent from the presented response format. One latent class often exhibits ERS. Another one exhibits MRS when there are few response categories (e.g., 4 options) but ordinary scale usage when the number of response categories is increasing up to 6 options (Eid and Rauber, 2000; Meiser and Machunsky, 2008).
2. Several kinds of ISU can occur simultaneously. For example, Baumgartner and Steenkamp (2001) reported about high correlations for ERS with DARS and ERS with ARS (r_ERS, DARS_ = 0.41, r_ERS, ARS_ = 0.59) and Weijters et al. (2010) confirmed these findings (r_ERS, DARS_ = 0.62, r_*ERS, ARS*_ = 0.72). That means that within a questionnaire, one may select extreme categories for an item set and dis-/agree with other items regardless of their content. Furthermore, particularly people with ERS are also inclined to reduce the presented response format to a few subjectively meaningful categories (Eid and Rauber, 2000; Meiser and Machunsky, 2008; Wu and Huang, 2010).
3. ISU depends on the trait, the population and context (Kieruj and Moors, 2013).

### Psychometric quality of data

High reliability and validity scores are often interpreted to reflect adequacy of the response format (Cox, 1980). For response formats with 2–10 options, the increase of the number of response categories of 2–6 categories only leads to an increase in reliability and convergent validity measured by a heterotrait-monomethod correlation (Lozano et al., 2008; Maydeu-Olivares et al., 2009; Culpepper, 2013). However, ISU is responsible for up to 25% of score variability (Wetzel and Carstensen, 2015) and can thereby make a contribution to the artificial increase of reliability (Weather et al., 2005; Jin and Wang, 2014). A separate assessment of true trait variance and response style variance is necessary to obtain an unbiased reliability measure. Chang (1994) demonstrated that a six-point rating scale contains a higher proportion of response style variance compared to a four-point scale, thus reducing psychometric quality. Weather et al. (2005) explained that particularly respondents with limited cognitive resources (e.g., discriminative capacity) react with intensive ISU on a long response format. Another important finding is that the reliability of homogeneous scales is less affected by the number of response categories (Weng, 2004). In general, a response format with 4–6 or seven response categories is considered optimal with regard to psychometric quality (Chang, 1994; Weng, 2004; Lozano et al., 2008; Culpepper, 2013). However, in large scale panel studies an 11-point scale and few items with diverse aspects of job are widely used and considered as golden standard of satisfaction assessment. To our knowledge, it has not been analyzed whether this response format is appropriate or produces ISU. Given the results of previous studies it is likely that there are individual differences in response style use. There may be individuals being overwhelmed by 11 categories, whereas other people might not have any problem with such a large number of response categories.

### The mixed item response theory approach for polytomous data

A suitable alternative for examining the adequacy of a response format is the mixed IRT approach, which allows modeling the response process on the level of single items and categories, as well as the exploration of heterogeneous scale usage. Mixed IRT models such as the mixed partial credit model (mPCM; Rost, 1997) can be applied to investigate a number of scale characteristics. The focus might lie on (a) heterogeneity of scale usage (e.g., Eid and Rauber, 2000); (b) proper usage of certain response categories (e.g., Carter et al., 2011); (c) adequately representing the continuity of a trait by the response format (e.g., Meiser and Machunsky, 2008), and on (d) stability of scale usage across items, subscales or different scales (e.g., Wetzel et al., 2013).

The number and size of latent subgroups are unknown and result by applying a mixed IRT model. The qualitative differences between subgroups in scale usage are detectable from the interpretation of subgroup specific item parameters and item profiles (Rost, 1997). Different types of ISU can be distinguished (e. g., actually, avoided' categories, usage of response styles, or socially desirable responding; see Eid and Zickar, 2007; Wu and Huang, 2010; Wetzel et al., 2013). Further, the resulting subgroup-specific latent trait values of respondents can be estimated. In contrast to raw total scores, those are adjusted to subgroup-specific scale usage and can be used to accurately compare individuals within and across latent subgroups (Eid and Rauber, 2000).

While the mPCM has been widely applied in the area of personality scales (e.g., Eid and Zickar, 2007; Maij-de Meij et al., 2008; Meiser and Machunsky, 2008; Wetzel et al., 2013), systematic research on how job satisfaction can be measured appropriately using this approach is still lacking. In the current paper, we will focus on evaluating the appropriateness of the long response format (11-point rating scale) that is used for assessing different aspects of job satisfaction in the HILDA survey. Because we assume that items will differ in their discriminating power, we will apply a mixture distribution IRT model with varying discrimination parameters of items to test whether there are subgroups that use the response format in different ways.

## Materials and methods

### Sample

We used data from the first wave (collected in 2001) of the HILDA survey. The HILDA survey is Australia's nationally representative household-based panel study. The data collection is primarily focused on subjective well-being, income and welfare, family formation, and labor market dynamics. The survey is conducted by the Melbourne Institute of Applied Economic and Social Research, from which the license for the data set can be obtained (Summerfield et al., 2015). While the general sample of the first wave consists of 13969 individuals, a subsample of 7036 subjects was obtained by the following inclusion criteria: A minimum age of 18, paid employment, and no missing values for the items on job satisfaction^1^. This sample consists of about half women (47.1%). The overall mean age is 39.2 years (*SD* = 11.48, *max* = 82). Concerning the level of education, 57.6% have at least a graduate degree. Most subjects are employees (93%) and most are working full time (73.7%).

### Measures

#### Job satisfaction (JS)

The HILDA survey includes 5 items on satisfaction with various aspects of the current job: total pay, job security, work itself, working hours and flexibility to balance work and non-work commitments. The response format consisted of an 11-point rating scale (0 = *totally dissatisfied* and 10 = *totally satisfied*).

#### Predictor variables

***Job position*** (JP) is a single item measure. The eight categories of the original variable were regrouped into the following hierarchical work levels: Specialists and executive staff (Level 1), administrators (Level 2), staff of the service sector such as machinery operators, drivers, and so forth (Level 3).

***Organization size*** (OS) is derived from an item on the number of persons employed in the organization of respondents. We defined an organization with less than 20 persons as small, with 20–200 persons as medium-size, and with more than 200 persons as a large one.

***Job Characteristics*** (JC) were measured with 10 items. Respondents evaluated their psychosocial work conditions using a seven-point rating scale from 1 (*strongly disagree*) to 7 (*strongly agree*). Based on an exploratory factor analysis, we reduced the 10 items to four aspects of working conditions: (1) *stress* (e.g., “My job is more stressful than I had ever imagined.”), (2) *security* (e.g., “The company I work for will still be in business 5 years from now.”), (3) *autonomy* (e.g., “I have a lot of say about what happens on my job.”), and (4) *skills* (e.g., “I use many of my skills and abilities in my current job.”) See Part A of the supplementary material for more details.

***The importance of employment and work situation*** is measured with one item using an 11-point rating scale from 0 (*the least important thing*) to 10 (*the most important thing*). Further single-item measures were ***the tenure in respondents' current occupation*** (in years), and ***total financial year income*** (AUD$ in thousands).

### Statistical analyses

#### Non-technical introduction into the IRT models

First, we will present a general unidimensional logistic model for ordered categorical data—the generalized partial credit model (GPCM; Muraki, 1992). We then describe a more general model incorporating latent subgroups, the mixed distribution generalized partial credit model (mGPCM; Von Davier and Yamamoto, 2004) as well as its more parsimonious variant—the restrictive mixed generalized partial credit model (rmGPCM).

##### The generalized partial credit model

The GPCM (Muraki, 1992) extends the partial credit model (PCM; Masters, 1982) to include item-specific discrimination parameters. Thus, the GPCM contains two kinds of parameters that link the manifest item responses to the continuous latent continuum: Item-specific threshold parameters τ_*is*_ that locate the “skip” between two adjacent categories *x* − 1 and *x*, and item-specific discrimination parameters δ_*i*_ that characterize the discriminating power of an item. The responses are modeled as probabilities *P*_*vix*_(θ) for category *x* (*x* ∈ {0,…, *m*}) of a polytomous item *i* given the latent trait score θ_*v*_ of an individual *v* and the both kinds of item parameters:

(1) $$\begin{array}{r} P_{vix}\left(\theta \right)=\frac{\exp\left[\sum_{s\;=\;0}^{x}\delta_{i}\left(\theta_{v}-\tau_{is}\right)\;\right]}{\sum_{c\;=\;0}^{m}\exp\left[\sum_{s\;=\;0}^{c}\delta_{i}\left(\theta_{v}-\tau_{is}\right)\right]}\\ \text{with }\delta_{i}>0\text{ and}\sum_{s\;=\;0}^{0}\delta_{i}\;\left(\theta_{v}-\;\tau_{is}\right)\;\equiv 0\text{ for all }i. \end{array}$$

**Figure 1:**
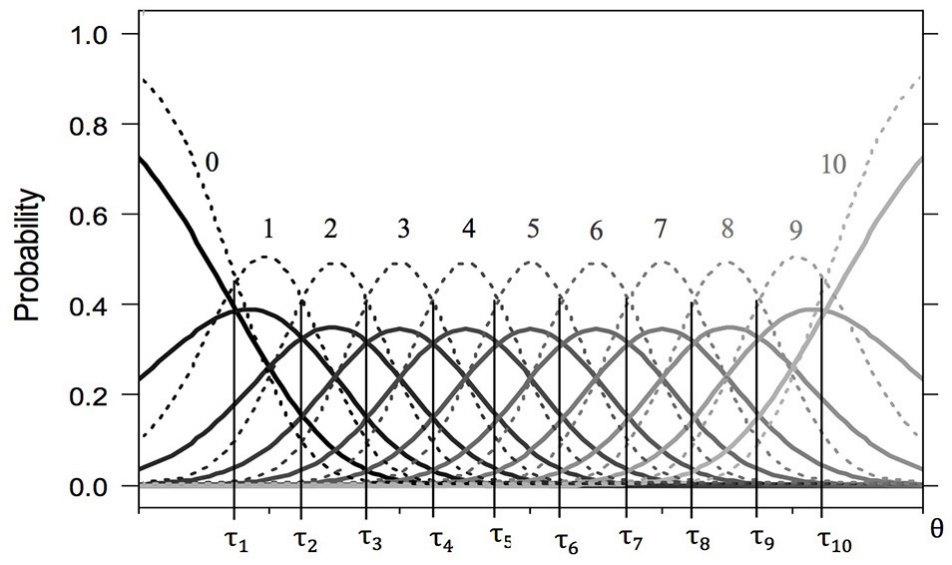
**Category characteristic curves for two fictitious items with 11 response categories (solid lines for item 1a and dashed lines for item 1b)**. Both items share the same ordered threshold parameters (τ_1_ = − 2.25, τ_2_ = −1.75, τ_3_ = −1.25, τ_4_ = −0.75, τ_5_ = −0.25, τ_6_ = 0.25, τ_7_ = 0.75, τ_8_ = 1.25, τ_9_ = 1.75, τ_10_ = 2.25), but differ in their discrimination parameters (δ_*a*_ = 1.50, δ_*b*_ = 3.00).

These response probabilities can be depicted as category characteristic curves (CCCs). Figure 1 shows the CCCs of two fictitious items with 11 response categories (*x* = 0, …, 10) that differ only in their discrimination parameter. For both items, the response probability for the first category is monotonically decreasing. With increasing latent trait value, selecting the first category becomes less likely. In an analogs manner, the probability for the last category is monotonically increasing—selecting this category becomes more likely with increasing θ value. The CCCs of all remaining categories are unimodal. The intersections of CCCs of adjacent categories *x* − 1 und *x* are represented by threshold parameters τ_*is*_. In general, there are *m* − 1 threshold parameters for each item. Thresholds are placed on the same scale as the latent continuum θ. The lower the threshold, the easier it is to choose the higher of the categories given the latent trait value θ_*v*_. The additional item-specific discrimination parameters δ_*i*_ that distinguish the GPCM from the PCM also affect response probabilities *P*_*vix*_(θ) in a particular category *x* of an item *i* at the latent trait values θ_*v*_. The higher the discrimination parameter, the steeper and narrower are the CCCs. In Figure 1, the dashed lines represent CCCs of an item with higher discrimination parameter compared to the solid lines.

##### The mixed generalized partial credit model

The mGPCM (Von Davier and Yamamoto, 2004) is an extension of the GPCM and assumes the existence of a priori unobserved subpopulations. It is defined by the following equation:

(2) $$\begin{array}{r} P_{vix}\left(\theta \right)=\overset{G}{\underset{g\;=\;1}{\sum }}\pi_{g}\frac{\text{ exp}\left[\sum_{s\;=\;0}^{x}\delta_{ig}\left(\theta_{vg}-\;\tau_{isg}\right)\right]}{\sum_{c\;=\;0}^{m}\text{exp}\left[\sum_{s\;=\;0}^{c}\delta_{ig}\left(\theta_{vg}-\tau_{isg}\right)\right]}\\ \text{with }\sum_{g\;=\;1}^{G}\pi_{g}=\;1,E\;\left(\theta_{vg}\right)=0\text{ for all }g,\\ \sum_{s\;=\;0}^{0}\delta_{ig}\;\left(\theta_{vg}-\;\tau_{isg}\right)\equiv 0\text{ for all }i\text{ in all }g. \end{array}$$

Each parameter of the mGPCM obtains an additional index *g* (*g* ∈ {1,…, *G*}), which indicates a latent subgroup. π_*g*_ (0 < π_*g*_ < 1) is the size of latent subgroup *g*. The number of latent subgroups *G* is no a model parameter but is determined by comparing models with a different number of classes by means of goodness-of-fit statistics. In the mGPCM there are subgroup-specific threshold and discrimination parameters. Therefore, the CCCs differ between latent subgroups and can be used to identify peculiar scale usage patterns within a homogeneous subgroup.

##### Restricted model version

Compared to the mGPCM, the rmGPCM assumes equal discrimination parameters across latent subgroups (but not items):

(3) $$\begin{array}{r} P_{vix}\left(\theta \right)=\overset{G}{\underset{g\;=\;1}{\sum }}\pi_{g}\frac{\text{ exp }\left[\sum_{s\;=\;0}^{x}\delta_{i}\;\left(\theta_{vg}-\;\tau_{isg}\right)\right]}{\sum_{c\;=\;0}^{m}\text{exp}\left[\sum_{s\;=\;0}^{c}\delta_{i}\;\left(\theta_{vg}-\tau_{isg}\right)\right]}\\ \text{with }\sum_{g\;=\;1}^{G}\pi_{g}=1,E\;\left(\theta_{vg}\right)=0\text{ for all }g,\\ \sum_{s\;=\;0}^{0}\delta_{ig}\;\left(\theta_{vg}-\;\tau_{isg}\right)\equiv 0\text{ for all }i\text{ in all }g. \end{array}$$

By contrast with the mGPCM, discrimination parameters δ_*i*_ lack index *g*. Moreover, in the mPCM these discrimination parameters are constrained to be equal across items (for details on the mPCM see Carter et al., 2011).

In our study, we will start with an application of the more parsimonious rmGPCM on the five JS items, because we suspect that the mGPCM (with item-specific discrimination parameters within latent subgroups) is too complex to fit well. We first determine the number and size of the latent subgroups by selecting the model solution of the rmGPCM that fits the data best. In the modeling process, we will compare the best solution of the rmGPCM to the more restrictive mPCM and then to the more general mGPCM. These model comparisons would reveal whether including discrimination parameters in a model improves the model-data fit. Finally, we will try to explain the assignment of individuals to subgroups from the best fitted model solution by means of socio-demographic factors and job-related variables.

#### Estimation

For estimating the rmGPCM (as well as above-named model variants), the Latent GOLD 5 software package was used (Vermunt and Magidson, 2013). Here, the marginal maximum likelihood function (MML) is maximized using an EM algorithm initially, switching to the Newton-Raphson method in the end. The number of iterations was set to 8000 and 600, respectively, and 100 sets of starting values were used (see Part B of the supplemental material for the syntax).

#### Model fit

In the first step, the adequate number of classes was determined by comparing rmGPCMs with one to five classes with regard to the consistent Akaike information criterion (CAIC; Bozdogan, 1987) which is suitable for comparison of IRT mixture models with varying number of subpopulations (Cho, 2013). The class solution with the lowest CAIC value indicates the preferable model. Additionally, in order to test whether the expected frequencies of response patterns in the selected model deviated significantly from the observed ones in the empirical data, we calculated parametric bootstrapping *p*-values for the Pearson and Cressie-Read χ^2^ goodness-of-fit statistics using 500 bootstrapping samples (default). In a second step, we tested whether model fit was improved by estimating (a) the more parsimonious mPCM, which assumes equal discrimination parameters across items, or (b) the more general mGPCM, which assumes item-specific discrimination parameters within classes. These models were compared to the rmGPCM by conducting bootstrapping χ^2^-difference tests. A significant test result indicates a better fit of the more complex model.

#### Exploring scale usage

**Figure 2:**
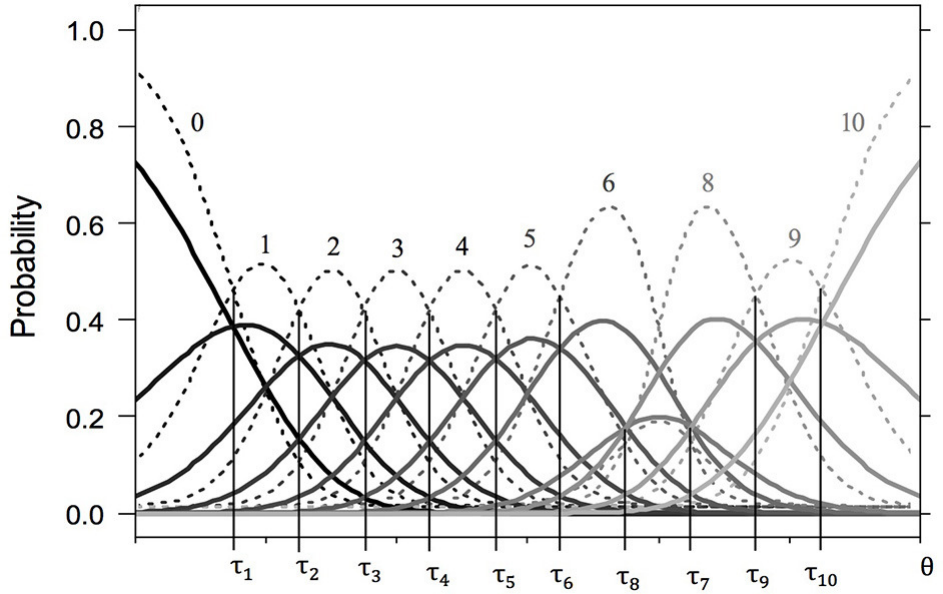
**Category characteristic curves for two fictitious items with 11 response categories (solid lines for item 2a and dashed lines for item 2b)**. Both items share the same partly disordered threshold parameters (τ_1_ = − 2.25, τ_2_ = −1.75, τ_3_ = −1.25, τ_4_ = −0.75, τ_5_ = −0.25, τ_6_ = 0.25, τ_7_ = 1.25, τ_8_ = 0.75, τ_9_ = 1.75, τ_10_ = 2.25), but differ in their discrimination parameters (δ_*a*_ = 1.50, δ_*b*_ = 3.00).

Mapping the item parameters and CCCs to scale usage of the best-fit model, the following three aspects are important: (1) Ordered thresholds mean that all given response categories are present on the latent continuum in ascending order. The items in Figure 1 represent the ordinary scale usage of an item, in that the thresholds are ordered and equidistant. Unordered thresholds often indicate avoided categories (Wetzel and Carstensen, 2014). In Figure 2, the order of τ_*i*8_ and τ_*i*7_ is reversed, indicating a complete overlap of the CCC pertaining to category *x* = 7 by the CCCs of categories *x* = 6 and *x* = 8. Apparently, this category is avoided. While a subgroup may exhibit ordered thresholds, another one may be characterized by the omission of certain categories. (2) The distance between adjacent thresholds represents the width of the latent category. The wider is the distance between thresholds, the larger is the range on the latent continuum represented by this category. Subgroups may also differ in this measure, with an ERS group characterized by very wide extreme latent categories. (3) Higher discrimination parameters lead less overlap between CCCs. Also, response probabilities then change more rapidly with increasing latent trait value.

#### Predicting class membership

Because the JC subscale scores showed a non-ignorable amount of missing data (8.5–9.4%) multiple imputation was applied (Enders, 2010). Following recommendations by Graham et al. (2007), we generated 20 data sets with missing values on JC subscales replaced by the means of a sequential regression method (as implemented in IBM SPSS Statistics package v23, IBM Corp., Armonk, NY, USA). The imputation model included all predictor variables, latent class membership and estimated person parameters in job satisfaction gained from the best class-solution of the mixed IRT model as well as personality traits such as conscientiousness that predicted the missingness (see Part C of the supplementary material for details on the missing analysis). The following analysis was automatically performed on the 20 generated data sets and results were subsequently aggregated. Class membership was predicted in a multinomial logistic regression model. Classification inaccuracy of the rmGPCM was taken into account by using the adjusted three-step method proposed by Vermunt (2010) that is implemented in Latent GOLD 5.0. For categorical predictors (e.g., job position, organization size) sets of dummy variables were built. To reduce the number of dummy variables, the categories of original predictor variables were regrouped as described above.

## Results

### Descriptive analysis

Table 1 gives descriptive statistics for the JS items. The relative frequencies demonstrate that response categories in the lower part of the response format are underrepresented. In particular, the two lowest categories were chosen by less than 2% of the sample. The category chosen most frequently was either 8 (*total pay, work itself*, *working hours* aspects) or 10 (*job security, flexibility*).

**Table 1:** **Descriptive statistics for aspects of job satisfaction**.

**Item label**	**Statistics**		**Relative category frequencies**										
	***M***	***SD***	**0**	**1**	**2**	**3**	**4**	**5**	**6**	**7**	**8**	**9**	**10**
Total pay	6.73	2.41	1.9	1.6	3.2	4.9	5.1	10.8	10.0	18.5	21.4	9.7	12.8
Job security	7.72	2.50	1.9	1.5	2.5	2.9	2.9	7.1	4.6	10.0	18.6	17.6	30.8
Work itself	7.67	2.06	0.5	0.7	1.4	2.4	2.6	7.3	7.7	15.2	23.6	17.5	21.1
Working hours	7.14	2.35	1.0	1.4	2.7	3.9	4.3	10.8	8.7	15.1	20.8	13.3	18.0
Flexibility	7.36	2.60	2.0	1.7	3.2	3.8	3.8	8.5	6.3	11.6	18.0	15.0	26.0

### Determination of the number of latent classes

All estimated models reached convergence. It took between 2 and 252 iterations in EM algorithm and 2 to 11 iterations in Newton-Raphson algorithm. Goodness-of-fit statistics for the rmGPCM with one to five classes can be found in Table 2. The three-class rmGPCM indicated the best relative model fit (CAIC_rmGPCM−3_ = 137637 is the lowest). Also, with respect to absolute fit, the three-class rmGPCM fitted the data very well (*p* > 0.05 for bootstrapped Pearson and Cressie-Read χ^2^ statistics).

**Table 2:** **Goodness-of-Fit statistics for the rmGPCM and competing models**.

**Model**	***N***_***par***_	**LL**	**CAIC**	**Pearson *p*-Value**	**CR *p*-Value**	**BV**	**Extr. τ_*isg*_**	**Extr. SE**	**Bootstrapped χ^2^-Difference Test (*df*) *p*-Value**
**rmGPCM**									
1 class	55	−69917	140376						
2 classes	107	−68382	137819						
3 classes	159	−68035	**137637**	0.99	0.99	0	1	7	
4 classes	211	−67877	137833						
5 classes	263	−67736	138065						
mPCM 3 classes	155	−68173	137873			0	2	5	rmGPCM vs. mPCM (3 cl): 272.81 (4) < 0.001
mGPCM 3 classes	169	−68009	137684			2	98	7	mGPCM vs. rmGPCM (3 cl): 51.87 (10) *p* = 0.94

*N_par_: the number of model parameter. LL, Log-Likelihood; CAIC, Consistent Akaike's Information Criterion; Pearson p-Value, the bootstrapped p-value corresponding to the Pearson χ^2^ goodness-of-fit statistic; CR p-Value, the bootstrapped p-value corresponding to the Cressie-Read χ^2^ goodness-of-fit statistic; BV, boundary values; Extr. τ_isg_, the number of threshold parameters larger than |4|. Extr; SE, the number of extreme standard errors of item parameters; (Extreme standard errors are defined as values five times larger than the most frequently occurring standard errors in the estimated model. Here, larger than 1.5)*.

*The lowest CAIC is marked in boldface*.

The three-class rmGPCM was then compared to the three-class mPCM and the three-class mGPCM. The three-class rmGPCM shows a better fit to the data than the mPCM (lower CAIC, Δχ^2^(4) = 272.81; bootstrapped *p* < 0.001). The rmGPCM and the mGPCM demonstrated no statistically relevant differences in their data fit (slight difference in CAIC values; Δχ^2^(10) = 51.87, bootstrapped *p* = 0.94), hence we accepted the more parsimonious three-class rmGPCM. More details for class solutions of the mPCM, rmGPCM and mGPCM are provided in Part D of the supplementary material.

### Subgroup specific scale usage

**Figure 3:**
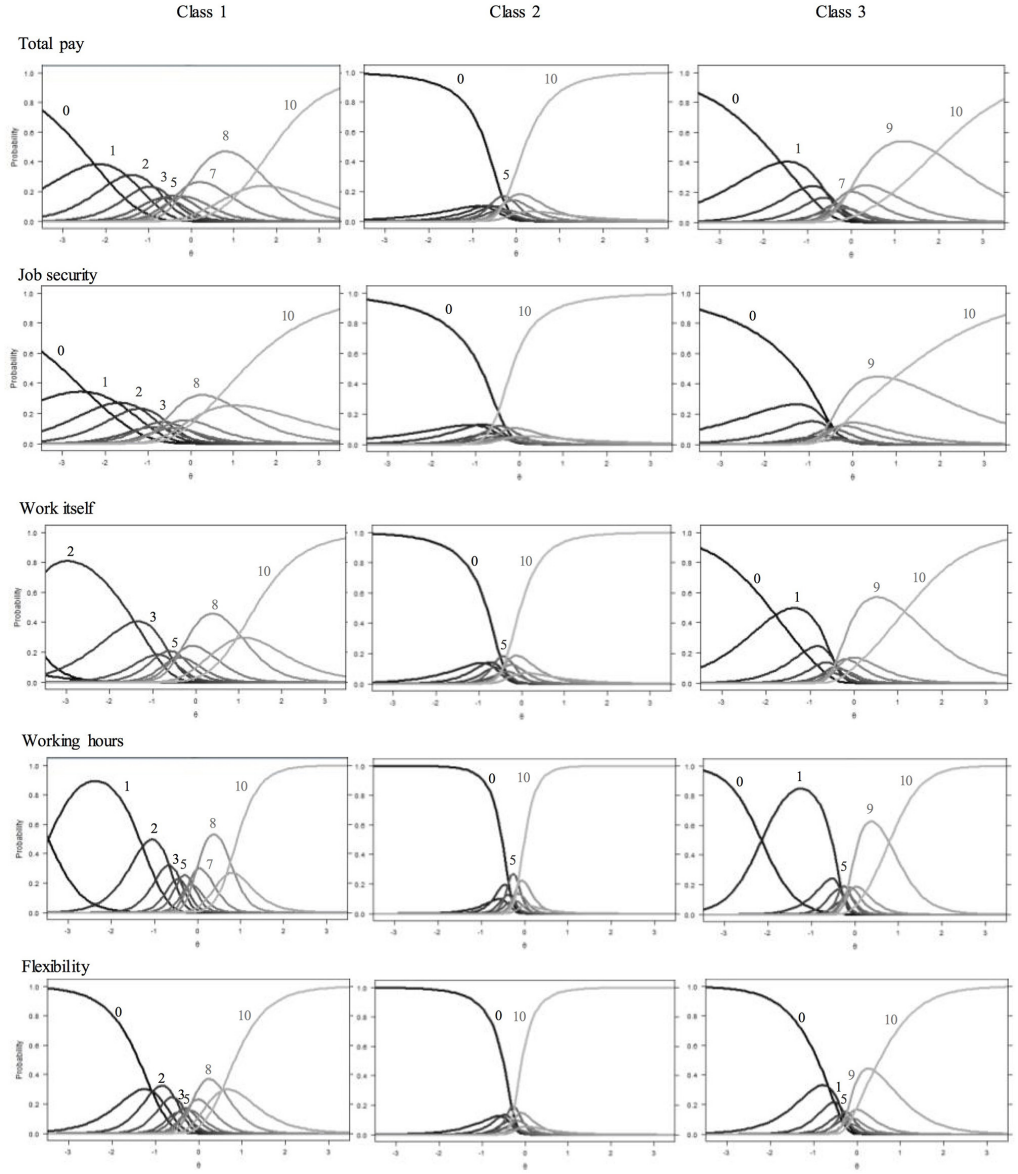
**Category characteristic curves for aspects of job satisfaction in the three latent classes**. (A number indicates the value of a category which has the highest response probability on a certain segment of the latent continuum).

Based on the rmGPCM-3 individuals were first assigned to latent classes by using their largest class assignment probability value. To evaluate the accuracy of classification, the mean assignment probability for each latent class was calculated. It can be considered as good and equals to 0.79 for the first class (as ordered by size, π_1_ = 0.40), to 0.85 for the second class (π_2_ = 0.33), and to 0.76 for the third class (π_3_ = 0.27). Table 3 presents the class-specific item parameters and the corresponding robust standard errors of the three-class rmGPCM. The category characteristic curves are shown in Figure 3. The classes differ with regard to scale usage in the following way: While in the first (largest) class, at least half of the thresholds (5 to 8, depending on the specific item) are in the order expected given the response format, this only holds true for 2 or 3 thresholds in the second class. The third (smallest) class can be placed between these two with 4 to 6 correctly ordered thresholds. This implies major deviations of class-specific response patterns from the ordinary scale usage in all classes. More evidence for presumed class-specific scale usage can be drawn from the difference between adjacent thresholds, which are far from equidistant (range_class1_ [0.28, 1.27], range_class2_ [0.67, 2.00], range_class3_ [0.37, 1.62]). Taken together, the most refined differentiation between response categories can be expected in the first class, a moderate one in the third class, and the crudest in the second class. We will now look more closely at the scale usage within each class.

**Table 3:** **Latent class specific item parameters from the rmGPCM with three class solution**.

**Item label**	**δ_*i*_**	**τ_*i*1*g*_**	**τ_*i*2*g*_**	**τ_*i*3*g*_**	**τ_*i*4*g*_**	**τ_*i*5*g*_**	**τ_*i*6*g*_**	**τ_*i*7*g*_**	**τ_*i*8*g*_**	**τ_*i*9*g*_**	**τ_*i*10*g*_**
**CLASS 1 (π_1_ = 0. 40)**											
Total Pay	1 (–)	−2.27 (1.91)	−1.52 (0.44)	−0.86 (0.22)	−0.41 (0.14)	−0.64 (0.12)	−0.25 (0.10)	−0.46 (0.08)	−0.10 (0.08)	1.92 (0.26)	1.16 (0.38)
Job security	0.71 (0.04)	−2.43 (1.63)	−1.72 (0.47)	−1.18 (0.24)	0.04 (0.16)	−1.16 (0.15)	−0.06 (0.13)	−0.84 (0.11)	−0.99 (0.09)	0.95 (0.14)	0.29 (0.14)
Work itself	1.27 (0.08)	2.44 (8.30)	−5.71 (6.66)	−1.42 (0.57)	−0.45 (0.23)	−0.83 (0.16)	−0.30 (0.12)	−0.53 (0.09)	−0.36 (0.07)	1.08 (0.19)	0.95 (0.30)
Working hours	2.58 (0.24)	−3.47 (2.21)	−1.28 (2.02)	−0.66 (0.33)	−0.41 (0.19)	−0.41 (0.14)	−0.11 (0.11)	−0.23 (0.09)	−0.03 (0.09)	0.86 (0.36)	0.67 (0.49)
Flexibility	1.76 (0.15)	−1.08 (0.96)	−1.06 (0.53)	−0.56 (0.23)	−0.22 (0.17)	−0.48 (0.16)	−0.17 (0.12)	−0.33 (0.09)	−0.16 (0.08)	0.56 (0.13)	0.53 (0.22)
**CLASS 2 (π_2_ = 0. 33)**											
Total Pay	1 (−)	0.98 (0.29)	−0.77 (0.31)	−0.52 (0.22)	−0.08 (0.20)	−1.22 (0.16)	0.61 (0.15)	−0.78 (0.14)	−0.24 (0.10)	1.24 (0.18)	−1.89 (0.18)
Job security	0.71 (0.04)	1.11 (0.29)	−1.00 (0.30)	0.10 (0.26)	−0.05 (0.27)	−1.79 (0.27)	1.47 (0.21)	−0.80 (0.24)	−1.35 (0.18)	0.98 (0.20)	−3.51 (0.19)
Work itself	1.27 (0.08)	0.22 (0.45)	−0.84 (0.47)	−0.44 (0.32)	−0.06 (0.32)	−1.39 (0.27)	0.25 (0.17)	−0.64 (0.16)	−0.49 (0.12)	0.74 (0.18)	−1.61 (0.17)
Working hours	2.58 (0.24)	0.12 (0.48)	−0.77 (0.48)	−0.26 (0.27)	−0.15 (0.28)	−0.77 (0.23)	0.21 (0.17)	−0.34 (0.18)	−0.29 (0.13)	0.56 (0.24)	−0.88 (0.23)
Flexibility	1.76 (0.15)	0.30 (0.30)	−0.57 (0.33)	−0.18 (0.27)	−0.16 (0.28)	−0.93 (0.23)	0.63 (0.23)	−0.69 (0.23)	−0.38 (0.14)	0.54 (0.19)	−1.38 (0.17)
**CLASS 3 (π_3_ = 0. 27)**											
Total Pay	1 (−)	−1.61 (0.50)	−0.60 (0.26)	−0.35 (0.22)	0.22 (0.25)	−0.83 (0.23)	−0.01 (0.19)	−0.85 (0.16)	−0.03 (0.13)	−0.11 (0.18)	1.93 (0.25)
Job security	0.71 (0.04)	−0.38 (0.35)	−0.28 (0.32)	0.61 (0.41)	−0.68 (0.41)	−1.43 (0.27)	1.33 (0.35)	−2.24 (0.35)	−0.33 (0.22)	−1.35 (0.22)	0.93 (0.14)
Work itself	1.27 (0.08)	−1.79 (0.74)	−0.47 (0.36)	−0.27 (0.37)	0.01 (0.43)	−0.84 (0.38)	−0.26 (0.26)	−0.68 (0.21)	−0.16 (0.17)	−0.76 (0.21)	1.15 (0.20)
Working hours	2.58 (0.24)	−2.15 (3.82)	−0.25 (0.32)	−0.31 (0.36)	−0.09 (0.38)	−0.59 (0.38)	−0.08 (0.22)	−0.24 (0.20)	−0.05 (0.19)	−0.30 (0.22)	0.79 (0.26)
Flexibility	1.76 (0.15)	−0.66 (0.36)	−0.39 (0.30)	−0.15 (0.33)	−0.18 (0.31)	−0.59 (0.30)	0.18 (0.24)	−0.36 (0.24)	−0.31 (0.19)	−0.45 (0.19)	0.40 (0.15)

*Threshold parameters τ_isg_ are transformed from differences between two adjacent categories parameters β_0ixg_−β_0ix−1g_, which were obtained in Latent GOLD Regression submodule, as follows τ_isg_ = −1 × (β_0ixg_−β_0ix−1g_) / δ_i_ (Vermunt and Magidson, 2006). Robust standard errors in brackets are calculated by Latent GOLD for the parameters β_0ixg_−β_0ix−1g_*.

#### Class 1

In this largest class, the first four thresholds are mainly ordered and rather equidistant. For the items *work itself* and *working hours*, this pattern is slightly altered because of extreme parameter estimates, most likely due to the low category frequencies. However, one can infer that in this class, person with a low latent trait level are expected to differentiate between the lower categories (*x* = 0 to *x* = 3). In the medium range of the latent continuum, thresholds are mostly unordered, and only categories *x* = 5 and *x* = 7—if at all—appear. Remarkable in the upper range of the latent continuum is the reversed order of thresholds 9 and 10, so that category *x* = 9 is completely covered by the wide neighboring categories. This shows that in this class the category *x* = 9 is generally avoided. Taken together, there is refined differentiation in the rating of low job satisfaction in this class, while above average and highly satisfied workers seem to decide only between two categories. Of the 11 categories presented in the manifest response scale, only 5–6 are represented on the latent continuum. Based on the particularities described above, we label the scale usage of this class *differential* response style (DRS).

#### Class 2

This class exhibits the most unordered thresholds and the category characteristic curves show the same pattern for all items: The two extreme categories (*x* = 0 and *x* = 10) dominate with high probabilities for a wide range on the latent trait level. For items *job security* and *flexibility*, these categories even intersect, indicating a dichotomous response pattern. For the remaining three items, there is a narrow medium range for which category *x* = 5 is most likely to be chosen. Because of this predominance of the two extreme categories, we label the scale usage of this second class *extreme* response style (ERS).

#### Class 3

Here, three to five categories appear to represent the full range of the latent continuum. For items *job security* and *flexibility*, the lowest category covers most of the lower half of the trait, while for the remaining items, there is also a considerable range in which the second category (*x* = 1) is most likely. In the upper half of all items, the two highest categories (*x* = 9, *x* = 10) dominate all remaining ones. There is a minimal range for the middle category for items *total pay* (*x* = 7) and *working hours* (*x* = 5). Taken together, the latent continuum is mostly reduced to four sections with roughly equal width. Because the dominating categories are the extreme ones, we label the scale usage of this third class *semi-extreme* response style (semi-ERS).

### Expected category frequencies for job satisfaction items in latent classes

**Figure 4:**
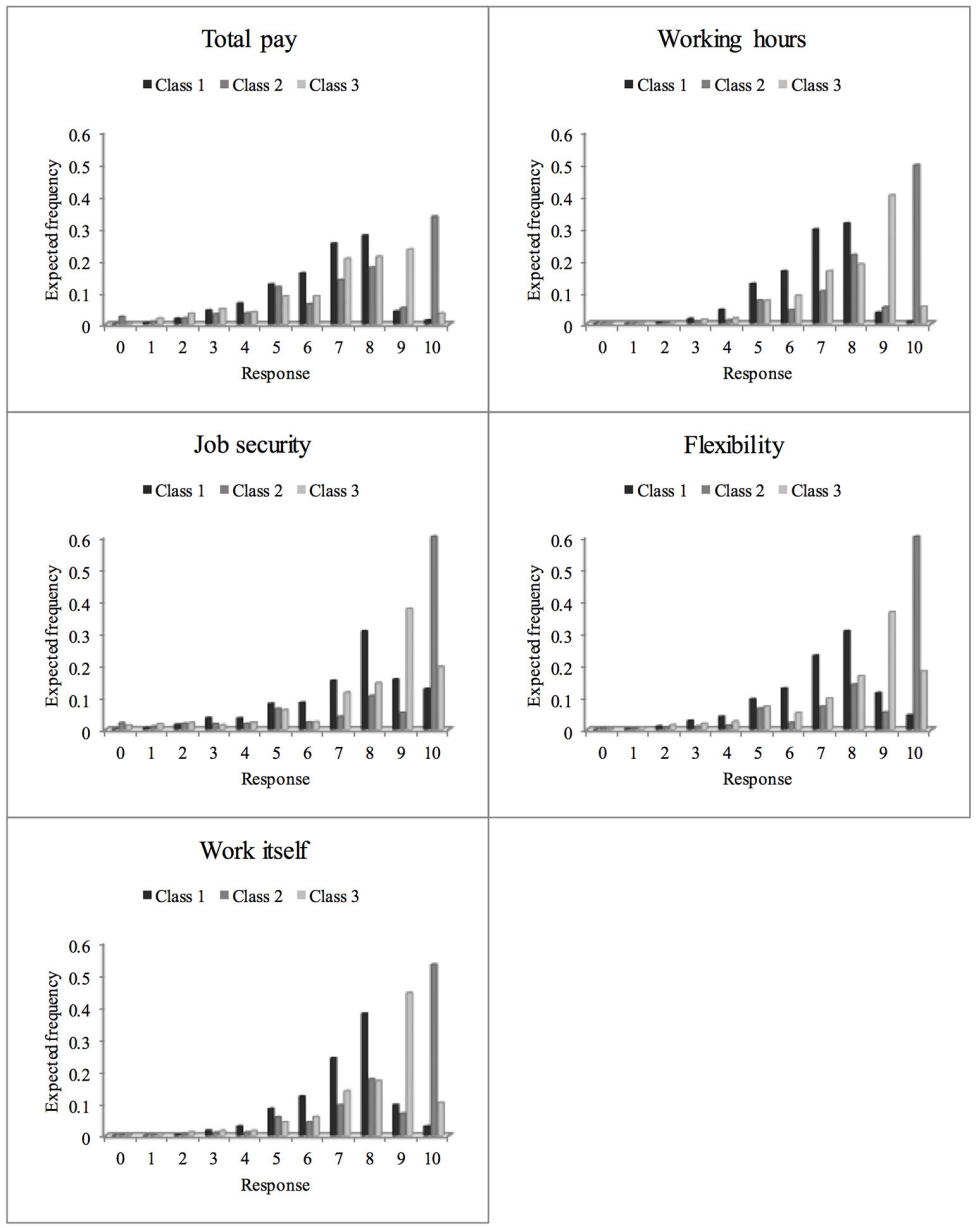
**Relative frequencies for the 11 response categories of the job satisfaction items expected on the basis of the rmGPCM-3 in latent classes**.

While evaluation of category characteristic curves allows identification of scale usage patterns, the distribution of the latent variable (job satisfaction) may also differ between latent classes. The expected category frequencies (see Figure 4) reflect both, differences in item parameters as well as differences in the distribution of latent variable between classes. Because all classes exhibit low expected frequencies in first five categories, the sample is quite satisfied on average. Differences between classes emerge in the upper categories. In the distribution of the first (DRS) class, the preference for category 8 becomes apparent, while for the ERS class, category 10 resp. category 9 for the semi-ERS class are expected to be most frequent.

### Predicting class membership

Results of the multinomial logistic regression for multiple imputed data sets can be found in Table 4. The assignment to the ERS class compared to the DRS class is more likely for female and part-time employees, with higher perceived job skills, job security, and greater importance of the job. Higher perceived job stress, on the other hand, makes an assignment to the ERS class less likely compared to the DRS class. In addition, assignment to the ERS class is more likely for employees in small organizations, with higher perceived autonomy, while it becomes less likely with higher education level and a high job position. The assignment to the semi-ERS class compared to the DRS class is more likely for female workers, workers in a part-time occupation, and for workers with high job skills, high job security, less job stress and a higher importance of the job. The assignment to the ERS class compared to the semi-ERS class becomes less likely with higher education level and increasing job stress. It becomes more likely for part-time employees and small organizations, as well as higher perceived autonomy, security, and importance of job.

**Table 4:** **Prediction of latent class membership by means of multinomial regression model**.

	**ERS class vs. DRS class**		**Semi-ERS class vs. DRS class**		**ERS class vs. Semi-ERS class**	
	***B*** **(SE)**	***e***^***b***^ **[95% CI]**	***B*** **(SE)**	***e***^***b***^ **[95% CI]**	***B*** **(SE)**	***e***^***b***^ **[95% CI]**
Constant	−7.16^***^ (0.49)		−4.53^***^ (0.52)		−2.63^***^ (0.53)	
Age	0.02^***^ (0.00)	1.02 [1.01; 1.03]	0.01 (0.01)	1.01 [1.00; 1.02]	0.01^**^ (0.01)	1.01 [1.00; 1.02]
Gender (female)	0.42^***^ (0.10)	1.53 [1.25; 1.86]	0.36^**^ (0.12)	1.44 [1.14; 1.81]	0.06 (0.11)	1.06 [0.85; 1.32]
Education level (>12 years)	−0.30^**^ (0.10)	0.74 [0.61; 0.90]	0.02 (0.12)	1.02 [0.81; 1.28]	−0.32^**^ (0.11)	0.73 [0.59; 0.90]
Income	−0.00 (0.00)	1.00 [1.00; 1.00]	0.00 (0.00)	1.00 [1.00; 1.01]	−0.00 (0.00)	1.00 [0.99; 1.00]
Tenure	0.00 (0.01)	1.00 [0.99; 1.01]	0.00 (0.01)	1.00 [0.99; 1.02]	−0.00 (0.01)	1.00 [0.99; 1.01]
Job position (Level 2)	−0.20 (0.13)	0.82 [0.63; 1.06]	−0.07 (0.16)	0.93 [0.68; 1.27]	−0.13 (0.14)	0.88 [0.67; 1.16]
Job position (Level 1)	−0.43^***^ (0.13)	0.65 [0.51; 0.83]	−0.27 (0.15)	0.76 [0.57; 1.03]	−0.16 (0.14)	0.85 [0.65; 1.11]
Part-time occupation	0.91^***^ (0.12)	2.49 [1.96; 3.16]	0.57^***^ (0.14)	1.76 [1.33; 2.33]	0.35^**^ (0.12)	1.41 [1.12; 1.79]
Organization size (small)	0.55^***^ (0.13)	1.72 [1.34; 2.22]	0.02 (0.14)	1.02 [0.77; 1.35]	0.53^***^ (0.14)	1.70 [1.29; 2.24]
Organization size (medium)	0.15 (0.13)	1.16 [0.90; 1.50]	0.09 (0.14)	1.09 [0.83; 1.44]	0.06 (0.14)	1.07 [0.81; 1.41]
Autonomy	0.16^***^ (0.03)	1.18 [1.10; 1.25]	0.03 (0.04)	1.03 [0.96; 1.11]	0.13^***^ (0.03)	1.14 [1.07; 1.22]
Skills	0.14^***^ (0.04)	1.15 [1.07; 1.23]	0.14^**^ (0.04)	1.15 [1.06; 1.25]	−0.00 (0.04)	1.00 [0.92; 1.08]
Security	0.28^***^ (0.04)	1.32 [1.23; 1.43]	0.11^*^ (0.04)	1.11 [1.02; 1.21]	0.17^***^ (0.04)	1.19 [1.10; 1.30]
Stress	−0.25^***^ (0.04)	0.78 [0.73; 0.83]	−0.08^*^ (0.04)	0.92 [0.86; 0.99]	−0.17^***^ (0.04)	0.85 [0.79; 0.91]
Importance of job	0.39^***^ (0.03)	1.47 [1.38; 1.58]	0.14^***^ (0.03)	1.15 [1.07; 1.22]	0.25^***^ (0.04)	1.29 [1.20; 1.39]
Job satisfaction	0.01^**^ (0.00)	1.01 [1.00; 1.01]	0.02^***^ (0.00)	1.02 [1.01; 1.03]	−0.01^***^ (0.00)	0.99 [0.98; 0.99]

*Reference group: the DRS class (left and middle part of the table), the semi-ERS class (right part of the table)*.

*R^2^ = 0.13 (Nagelkerke). The model indicates a significant improvement in the fit comparing to the baseline model: χ^2^ (32) = 877.82, p < 0.001*.

* *p < 0.05*,

** *p < 0.01*,

*** p < 0.001

Apparently, subjects with a high probability of being assigned to the DRS class are male and full-time employees with higher perceived job stress and a lower importance of the job. Subjects more likely assigned to the ERS class compared to the other two classes have a basic educational level, work part-time in small organizations and value their job as important. Both factors age and job satisfaction can hardly differentiate between the subgroups with different scale usage (the odds ratio are near to one).

## Discussion

The popularity of measuring job satisfaction with an 11-point rating scale in national panel surveys contrasts with the lack of empirical research on the adequacy of such a long response scale. This study aimed to fill this gap and evaluate how appropriate such a long response format is. The application of a restricted mixed general partial credit model on JS data from the HILDA Survey revealed severe drawbacks of an 11-point rating scale that we summarize in the next section. Afterwards, we will discuss the results of the multinomial logistic regression analysis that related the class-specific scale usage to covariates. Finally, we will discuss some implications and limitations of the study presented.

### Drawbacks of an 11-point rating scale

#### Low frequencies in certain categories

Explorative analysis showed the typical left-skewed distribution of the JS items, with very low frequencies in the first few categories. One can conclude that Australian employees are mostly satisfied with their jobs and hardly need to differentiate within the scale region pertaining to *dis*-satisfaction. The same can be observed for subgroup-specific distributions of JS items implied by the model.

#### Patterns of inappropriate scale usage

Applying the rmGPCM to JS items allowed us to identify three latent subgroups. About 40% of Australian employees were assigned to a class that avoids certain categories but differentiates reasonably among the remaining ones (up to six). In contrast, one third of the sample was assigned to a class with extreme response style, dichotomizing the scale into the two extreme categories. The remaining class exhibited a pattern with differentiation in the extremes: The two lowest categories and the two highest categories were used. In general, we found that none of latent subgroups differentiated between all of the 11 response categories presented in the HILDA Survey. Evidently, Australian employees evaluated aspects of their job satisfaction using only two to six response categories.

Overall, these results are consistent with previous research that has assessed ISU in short response formats (four to six categories). However, our results add important aspects to existing knowledge, in particular with respect to response scales with 11 categories. This study detected a very high proportion of subjects with ISU. In fact, all latent subgroups subjectively reduced the number of response categories. Some kind of ERS was detected for 60% of the sample, while the proportion of ISU is commonly estimated to involve about a third of subjects. We detected two latent subgroups that used different kinds of ERS. Previous studies, however, consistently reported one latent ERS subgroup. Whereas the combination of avoided categories and ERS has been previously observed (Eid and Rauber, 2000; Wu and Huang, 2010), the number of avoided categories was especially large in our study. The results revealed that one has to expect larger number of unused categories and different types of ERS the larger the number of response categories is.

#### Consequences for scale use

According to the results of our study, respondents use the scale in different ways. Therefore, comparing individuals using their total score might partly represent individual differences in response style and not differences in job satisfaction. It is important to note that more traditional psychometric methods are not able to detect these response style differences. For example, an exploratory factor analysis of the matrix of polychoric correlations of the five items indicated a one-factor solution (eigenvalues: 2.39, 0.83, 0.69, 0.65, 0.44). Furthermore, the psychometric quality of a scale might be overestimated when ISU is not considered. For example, the reliability coefficient of the JS measure after controlling ISU by means of rmGPCM-3 is lower (Rel_θ_v__ = 0.59) than the coefficient calculated on the basis of raw values of JS items that contain ISU variance (Cronbach's α = 0.67). According to the results presented one might get more valid results in studies aimed at explaining individual differences in job satisfaction by taking response styles into account. This could be done by assigning individuals to response style classes first and then analyzing interindividual differences in job satisfaction by taking the estimated person parameters.

### Explanation of class membership

The pattern of relevant predictors distinguishing between the latent classes (see Table 4) encourages to think of a typical member of the respective classes. As mentioned above, members of the DRS class tend to be male, working full-time with high perceived job stress and low personal job importance. In contrast to the DRS class, a basic educational level and a low job position are related to the ERS class. Members of the ERS evaluated their job conditions as positive (high job autonomy, suitable tasks with regard to qualifications, high job security, and low job-related stress). They tend to be female and working part-time, predominantly in small organizations. Similarly, members of the semi-ERS class are also more likely to be female and working part-time, but their educational level does not differ from members of the DRS class.

### Implications

For the population of Australian employees, a 6-point rating scale seems more adequate than the commonly used 11-point scale. We found that even in the differentiating class, only six of the given categories mapped well onto the latent trait. Experimental studies that systematically vary the number of categories are needed to gather more evidence for an optimal number of categories.

In general, mixed IRT models for polytomous data allow to evaluate two aspects of ISU: Avoided categories and response style. Moreover, different types of ISU within a single sample can be identified. The results provide a researcher with relevant information on the appropriateness of the response format that was administered. In the majority of mixed IRT studies so far, researchers have been using the mPCM (and its restrictive variants) for exploring ISU. In the present study, the rmGPCM exhibited a better fit to the data. Varying discrimination power of JS items seems a relevant item characteristic and should be taken into account. In contrast, using a model that is more general than necessary—the mGPCM-3—for the JS data is suboptimal: Class-specific discrimination parameters are redundant, make the model too complex and reduce the estimation accuracy of parameters (as indicated by many extreme parameters and standard error estimates). In general, the application of IRT models requires large sample sizes. If there are many parameters to estimate and the sample size is small, more restrictive models may be preferred over the rmGPCM due to estimation problems. Future simulation studies for mixed IRT models can help to clarify the optimal sample size required for functionally accurate estimation of parameters under various data conditions (e.g., number of items and response categories).

## Limitations

There were some minor estimation problems in the present application^2^ that are most likely due to the low observed frequencies in the lower response categories.

While with mixed IRT models for polytomous data one takes a typological approach toward modeling ISU, models of the dimensional framework (e.g., multidimensional IRT models, SEM) would be needed to disentangle effects of the response format and effects of dispositional ISU tendencies (independently of the measured trait and the number of categories). The mixed IRT approach is beneficial for exploring ISU patterns in data, whereas multidimensional IRT models and SEM appear to be superior in eliminating effects of ad hoc defined ISU from latent trait values (Wetzel et al., 2015).

In this study, mostly contextual factors and socio-demographic variables were available to predict the ISU classes. Job conditions were found to be the best predictors. However, the full regression model was only able to explain a small portion of the variability in classes (pseudo *R*^2^ = 13%). This re-emphasizes the need for research on causes of ISU. Future research should take into account the relative stability and trait-independence of some aspects of ISU (e.g., ERS) and include cognitive abilities (e.g., discriminative capacity) and associated dispositions (e.g., intolerance to ambiguity, decisiveness, impulsivity, social desirability). Because this is the first study that investigated ISU in job satisfaction data assessed by means of an 11-point response format, further studies are required to replicate our findings.

## Conclusions

The present study is the first one that investigated the appropriateness of a long response format (11-point rating scale) for assessing aspects of job satisfaction by exploring ISU. Three scale usage patterns were extracted for Australian employees. Two features of subgroup-specific ISU—(a) avoidance of several response categories in all latent classes and (b) preferred usage of semi-ERS and ERS in two qualitatively different latent classes—provided empirical evidence that the 11-point rating scale contains redundant categories and evokes usage of simplification strategies. Our findings show that a rating scale with 11 response categories is suboptimal for collecting high quality data on aspects of job satisfaction and provide essential clues for a better response format (e.g., 2–6 categories; no middle point).

## Author contributions

TK, CC, and ME substantially contributed to the conceptualization of this research, drafting and revising the manuscript, and approving the final version submitted.

## Funding

This paper uses unit record data from the Household, Income and Labor Dynamics in Australia (HILDA) Survey. The HILDA Project was initiated and is funded by the Australian Government, Department of Social Services (DSS) and is managed by the Melbourne Institute of Applied Economic and Social Research (Melbourne Institute). The findings and views reported in this paper, however, are those of the authors and should not be attributed to either the DSS or the Melbourne Institute.

### Conflict of interest statement

The authors declare that the research was conducted in the absence of any commercial or financial relationships that could be construed as a potential conflict of interest.

## Footnotes

^1^Proportion of cases with missing values on these items ranged from 0.1 to 0.3%.

^2^One extreme threshold parameter and a few large standard errors for threshold parameters, mainly for parameters of the DRS class. A similar picture gives the mPCM-3 (see Table 2).
