# Supplementary material for: Using a Mixed IRT Model to Assess the Scale Usage in the Measurement of Job Satisfaction
Source: Front Psychol. 2017 Jan 4;7:1998. doi: 10.3389/fpsyg.2016.01998 (PMC5209345; doi:10.3389/fpsyg.2016.01998)
Supplement: Supplementary file 1 [file Presentation1.pdf]

Supplementary Material

Using a Mixed IRT Model to Assess the Scale Usage in the Measurement of Job Satisfaction

Tanja Kutscher*, Claudia Crayen, Michael Eid

*** Correspondence:** Tanja Kutscher: tanja.kutscher@fu-berlin.de

Part A

Job Characteristics (JC)

The HILDA Panel Survey (wave 2001) includes 12 JC items on aspects of job quality. But the HILDA User Manual provides no information on evaluation of JC data. To examine the underlying structure of the JC measure, an explorative factor analysis (EFA) was conducted in Mplus software (available version 7, Muthen and Muthen, 2006) based on valid JC data of the analysis sample (*n* = 6302). We used the ML estimator. Factors were allowed to correlate (geomin rotation per default). The EFA detected four factors which eigenvalues are above 1 (eigenvalues: 2.85, 2.40, 1.61, 1.04, .90, .66, .58, .48, .43, .41, .36, .31). The standardized factor loadings of the four-factor solution are provided in table S1. Most JC items could be assuredly assigned to only one factor (loadings above |.40| on one factor and loadings less than |.20| on others). The exceptions are two items: the item *c* 'I get paid fairly for the things I do in my job.' which has low loadings on all factors and the item *g* 'My job is complex and difficult.' which loads equally high on factor 1 and factor 4. These items were not considered by creating subscales. Thus, four factor structure reflexes the following aspects of job quality derived from the item content: (1) job-related stress, (2) job security, (3) degree of freedom in action and in decision-making at work, and (4) requirements of applying and extending one's professional skills.

References

Muthén, L. K., and Muthén, B. O. (2012). *Mplus: statistical analysis with latent variables; user's guide version 7*. Muthen & Muthen, Los Angeles, California.

Table S1

Standardized factor loadings in the exploratory factor analysis for JC items

Item	EFA			
	1st factor	2nd factor	3rd factor	4th factor
a. My job is more stressful than I had ever imagined.	**.82***	.03*	-.02*	.04*
b. I fear that the amount of stress in my job will make me physically ill.	**.77***	-.03*	-.02*	-.05*
c. I get paid fairly for the things I do in my job.	-.21*	.16*	.14*	-.02
d. I have a secure future in my job.	.06*	**.90***	.04*	.00
e. The company I work for will still be in business 5 years from now.	-.03*	**.55***	-.10*	.10*
f. I worry about the future of my job	.13*	**-.49***	.00	.08*
g. My job is complex and difficult.	.37*	-.00	.08*	.48*
h. My job often requires me to learn new skills.	.01	-.03*	-.08*	**.82***
i. I use many of my skills and abilities in my current job.	-.02	.07*	.18*	**.56***
j. I have a lot of freedom to decide how I do my own work.	-.06*	-.01*	**.82***	.04*
k. I have a lot of say about what happens on my job.	.03*	.06*	**.80***	.04*
l. I have a lot of freedom to decide when I do my work.	.01	-.05*	**.70***	-.10*

Note: Factor loadings over |.40| are marked in bold. Items shown in gray are not assigned to any factors.

* $p < .05$.

Part B

Latent GOLD Script for Estimating the rmGPCM in Regression Submodule

```
options
   algorithm
      tolerance=1e-008 emtolerance=0.01 emiterations=8000 nriterations=600;
   startvalues
      seed=0 sets=100 tolerance=1e-005 iterations=200;
   bayes
      categorical=1 variances=1 latent=1 poisson=0;
   quadrature nodes=80;
   missing  excludeall;
   output
      parameters=first  // The first category parameter for all items is fixed to null.
      standarderrors=robust classification profile probmeans=posterior
      estimatedvalues=model iterationdetails identification
/* Estimated individual latent trait values and class assignment will be saved as a SAV
file. */
      outfile 'estimated person parameters.sav' classification;
 variables
      caseid id;
      dependent response; // 'response' consists of multiple responses of cases to all
items.
      independent itemnr nominal; // 'itemrt' is a nominal variable containing item IDs.
    latent
      theta continuous,
      class nominal 3; // '3' is the number of latent classes in the model.
 equations
      theta | class;  // Class-specific variance of the trait variable is freely
estimated.
      class <- 1;    // Sum of class proportions is fixed to 1.
/* The model equation below is in the form of a logistic regression model with 'response'
as a dependent variable and 'itemnr' as an independent variable.
In this equation the first term estimates differences in category parameters of two
adjacent categories, which can vary for all items in all classes. The second term in
addition to the equation in the last line fixes the discrimination parameter only of the
first item to 1.*/
      response <- (~diff) 1 | itemnr class + (L) theta | itemnr;
      L[1]=1;

// The mean of theta variable in each latent class is fixed to null per default.
```

Part C

Missing Analysis

For checking missing data mechanisms, we compared groups with missing values and valid values on JC subscales by the means of socio-demographic factors (e.g., age, gender, educational level, total financial year income), personality (e.g., the Big Five and personal control)[1], job-related variables (e.g., JC subscales, tenure in the current occupation, job importance), as well as two latent variables from the rmGPCM-3 application (e.g., latent class membership and estimated latent level of job satisfaction). To achieve this, we first built dichotomous variables for JS subscales (missing indicators). For continuous comparison variables we then conducted a series of independent-sample *t*-tests using each missing indicator as a group variable. Table S2 demonstrates *t*-test statistics and effect sizes (Cohen's standardized mean difference). For categorical variables, relative frequencies in the missing group as well as results of χ^2-test statistics are reported. The non-significant *t*- or χ^2-test statistics allow identifying variables with missing completely at random (MCAR). In contract, significant results and large effects indicate other missing data mechanisms such as missing at random (MAR) or missing not at random (MNAR) (see Enders, 2010).
Table S2 includes only comparison variables which produced significant test statistics.

References

Enders, C. K. (2010). *Applied Missing Data Analysis*. New York: Guilford Press.

Pearlin, L. I., and Schooler, C. (1978). The structure of coping. *Journal of health and social behavior*, *19*, 2-21.

Summerfield, M., Freidin, S., Hahn, M., Li, N., Macalalad, N., Mundy, L., et al. (2015). *HILDA User Manual–Release 14*. Melbourne, Australia: Melbourne Institute of Applied Economic and Social Research, University of Melbourne.

---

[1] The HILDA Survey Team provides the data on the five dimensions of personality (e.g., extraversion, agreeableness, conscientiousness, emotional stability, and openness to experience) for the first time at the 5th wave (in 2005) and for personal control scale (Pearlin and Schooler, 1978) which measures self-estimated extent of one's control over one's life circumstances at the 3rd wave (in 2003) (for details, see the HILDA User Manual – Release 14 by Summerfield et al., 2015). For this reason, we used these data only for handling missing values in predictor variables.

frontiers

Table S2

Comparison of groups with missing and valid values for JC subscales

	Comparison variable									
	Cons	Emot	PC	JS	Secu		Gender		Education	
							Male	Female	≤12 y.	> 12 y.
Indicator						MG				
Auto (*t*)	-4.57***	-1.95	-3.00[1]**	-3,07[1]**	-3.28**	%	9.3	7.5	9.5	7.7
(*d*)	-0.29	-0.13	-0.19	-2.65	-0.85	$\chi^2(df)$	6.94** (1)		6.71* (1)	
Secu (*t*)	-4.31***	-2.10*	-2.51[1]*	-2.72[1]**	-	%	10.0	8.7	10.8	8.4
(*d*)	-0.26	-0.13	-0.15	-2.14	-	$\chi^2(df)$	3.97* (1)		11.70** (1)	
Skills (*t*)	-4.53***	-1.85	-2.90[1]**	-3.16[1]**	-1.44	%	9.3	7.6	9.4	7.8
(*d*)	-0.29	-0.12	-0.18	-2.61	-0.36	$\chi^2(df)$	9.90** (1)		5.89* (1)	
Stress (*t*)	-4.37***	-1.86	-2.95[1]**	-3.25**	-1.14	%	9.4	7.6	9.6	7.8
(*d*)	-0.28	-0.12	-0.18	-2.67	-0.25	$\chi^2(df)$	7.51** (1)		7.06** (1)	

Note. Cons = Conscientiousness, Emot = Emotional stability, PC = Personal control, JS = estimated latent trait level of job satisfaction (scaled from 0 to 100), Auto = autonomy, Secu = security. *t* = T-Test Statistic. *d* = Standardized Mean Difference; $d < 0$ represents that the group with observed values had a higher mean, and $d > 0$ indicates that the persons with missings had a higher mean. MG = missing group.

[1] Welch Statistic.

* $p < .05$, ** $p < .01$, *** $p < .001$.

Part D

Goodness-of-Fit Statistics for the mPCM, rmGPCM, and mGPCM

Table S3
Goodness-of-Fit Statistics for the mPCM, rmGPCM, and mGPCM

Model	N_{par}	$N_{\text{iter.}}$ in EM	$N_{\text{iter.}}$ in NR	LL	BIC	CAIC	AIC	BV	Extr. τ_{isg}	Extr. SE
mPCM										
1 class	51	1	2	-70131	140714	140765	140364	0	0	0
2 classes	103	2	2	-68555	138023	138126	137317	0	1	1
3 classes	155	2	2	-68173	137718	137873	136655	0	2	5
4 classes	207	2	3	-67943	137720	137927	136301	0	3	8
5 classes	259	2	6	-67796	137887	138146	136110	0	16	22
rmGPCM										
1 class	55	2	2	-69917	140321	140376	139944	0	0	0
2 classes	107	2	2	-68382	137712	137819	136979	0	0	0
3 classes	159	2	4	-68035	**137478**	**137637**	136388	0	1	7
4 classes	211	45	9	-67877	137622	137833	136175	0	4	25
5 classes	263	252	5	-67736	137802	138065	135998	0	10	23
mGPCM										
1 class	55	2	2	-69917	140321	140376	139944	0	0	0
2 classes	112	2	3	-68376	137745	137857	136976	1	28	1
3 classes	169	2	11	-68009	137515	137684	136356	2	98	7
4 classes	226	2	14	-67818	137639	137865	136089	3	266	21
5 classes	283	182	nc	-67692	137891	138174	135950	-	-	-

Note. N_{par}: the number of model parameter. $N_{\text{iter.}}$ in EM: the number of iterations needed to reach convergence in EM algorithm. $N_{\text{iter.}}$ in NR: the number of iterations needed to reach convergence in Newton-Raphson algorithm. LL: Log-Likelihood. BIC: Bayesian information criterion. CAIC: Consistent Akaike's information criterion. AIC: Akaike's information criterion. Pearson *p*-Value: the bootstrapped *p*-value corresponding to the Pearson χ^2 goodness-of-fit statistic. CR *p*-Value: the bootstrapped *p*-value corresponding to the Cressie-Read χ^2 goodness-of-fit statistic. BV: boundary values. Extr. τ_{isg}: the number of threshold parameters larger than |4|. Extr. SE: the number of extreme standard errors of item parameters. (Extreme standard errors are defined as values five times larger than the most frequently occurring standard errors in the estimated model (here, larger than 1.5). nc: non-convergence.

The lowest BIC and CAIC are marked in boldface.
